# Evaluating the prognostic value of the stress index in trauma patients

**DOI:** 10.1016/j.heliyon.2024.e36884

**Published:** 2024-08-24

**Authors:** Pao-Jen Kuo, Ching-Ya Huang, Shiun-Yuan Hsu, Ching-Hua Hsieh

**Affiliations:** a Department of Plastic Surgery, Kaohsiung Chang Gung Memorial Hospital and Chang Gung University College of Medicine, Kaohsiung, 83301, Taiwan; b Department of Trauma Surgery, Kaohsiung Chang Gung Memorial Hospital and Chang Gung University College of Medicine, Kaohsiung, 83301, Taiwan

**Keywords:** Stress index (SI), Glucose, Potassium, Trauma, Mortality

## Abstract

**Background:**

The stress index (SI), defined as the serum glucose to potassium ratio, has emerged as a potential prognostic indicator in some patient populations. This study aims to evaluate the predictive value of SI on the trauma patients sustained by all trauma causes.

**Methods:**

A retrospective analysis was conducted on 20,040 adult trauma patients admitted to a single trauma center from January 1, 2009, to December 31, 2022. The SI was calculated according to the serum levels of glucose (mg/dL) and potassium (mEq/L) upon patients’ arrival to emergency room. The enrolled patients were stratified into two groups based on an optimal SI cutoff value determined by receiver operating characteristic (ROC) curve analysis. The association between SI and in-hospital mortality, as well as other clinical outcomes, was assessed using multivariate logistic regression, adjusting for potential confounders.

**Results:**

The mortality patients had a significantly higher SI (59.7 ± 30.6 vs. 39.5 ± 17.5, p < 0.001) than those who survived. The SI was identified as a significant independent predictor of mortality (odds ratio [OR] 4.65, 95 % confidence interval [CI]: 2.61–8.27, p < 0.001) in the multivariate analysis. In addition, patients in the high SI group (≥42.7) demonstrated significantly worse outcomes, including higher in-hospital mortality (7.5 % vs. 1.4 %, p < 0.001), longer hospital stays compared to the low SI group (<42.7).

**Conclusion:**

The SI serves as a simple and valuable prognostic tool in risk stratification of the trauma patients.

## 1 Introduction

Glucose levels detected in the emergency room play a crucial role in predicting outcomes and guiding management decisions for trauma patients. Admission blood glucose levels have been identified as an independent indicator of shock and mortality in multiply injured patients, aiding in the identification of those at higher risk [1]. Early hyperglycemia in trauma patients is crucial as it correlates with multiple organ failure, highlighting the significance of monitoring glucose levels for prognostic assessment and management strategies [2]. Admission blood glucose levels in trauma patients predict morbidity, intensive care unit, and hospital stay duration, independent of injury severity, age, and gender, emphasizing the significance of glucose monitoring in the emergency room [3]. Undiagnosed or poorly controlled diabetes in trauma patients underscores the importance of routine screening and intervention for improved glucose control to enhance long-term management outcomes [4]. Moreover, studies have shown that higher blood glucose levels are associated with a higher likelihood of positive findings in computed tomography in mild head trauma cases, with a cutoff value of 120 mg/dL being indicative of such cases [5]. Combining blood glucose evaluation with trauma scoring systems like the Revised Trauma Score has been found to enhance the accuracy of predicting clinical outcomes [6].

Potassium levels in trauma patients presenting to the emergency room hold significant clinical implications. Potassium levels in the emergency room are significant, as hyperkalemia incidence was 6.2 %, with 0.3 % severe cases, in trauma patients over a 10-year period [7]. Hyperkalemia, characterized by higher potassium levels, is common in trauma patients and can lead to adverse outcomes, including mortality and prolonged hospital stays [8,9]. It is very important to find and treat hyperkalemia as soon as possible because it can damage the heart's electrical system and need treatments like calcium salts and potassium-shifting agents [10]. Conversely, hypokalemia is also prevalent in trauma patients and is associated with increased mortality risk, adverse events, and longer hospital stays [11].

In patients with subarachnoid hemorrhage, the stress index (SI), also known as the serum glucose to potassium ratio (GPR), which is computed by dividing the serum glucose level by the serum potassium level upon arrival, was initially postulated to be correlated with plasma catecholamine levels [12,13]. The serum SI can be served for predicting the poor outcomes of patients with severe traumatic brain injury [14], acute intracerebral hemorrhage [15], ischemic stroke patients [16], or after aneurysmal subarachnoid hemorrhage [17]. SI has been proven to be helpful in predicting mortality and the indication for surgery in patients with isolated blunt thoracoabdominal trauma, with the SI exhibiting higher predictive capacities than the shock index [18]. Marini and Sein also talked about how SI can help with the first decisions about how to treat people with traumatic brain injuries. They linked this biomarker to how the patients did at 6 and 12 months and stressed how important it is when deciding whether to do surgery [19]. The predictive power of isolated SI for mortality and morbidity is greater than that of glucose and potassium levels [14,15,17,18]. Furthermore, studies have shown that SI can predict severe trauma, the need for damage control operations, and massive transfusions among trauma patients, aiding in early judgments for life-saving interventions [20].

These results highlight the capability of SI as a simple but practical technique for early prediction and treatment in some trauma patients, especially those with brain injuries [14,15]. This helps in quickly evaluating and categorizing these individuals according to their risk level. While previous research has explored the SI's utility in specific trauma scenarios such as traumatic brain injury or thoracoabdominal trauma, this study extends its application to a broader trauma population. The research seeks to determine whether the SI can serve as a simple yet effective tool for early risk stratification across various trauma types. By analyzing a large cohort of 20,040 adult trauma patients over a 14-year period, the study aims to establish an optimal SI cutoff value for predicting mortality and evaluate the prognostic value of the SI in trauma patients from all causes upon their initial presentation to the emergency department.

## 2 Materials and methods

### 2.1 Study population and data collection

**Fig. 1:**
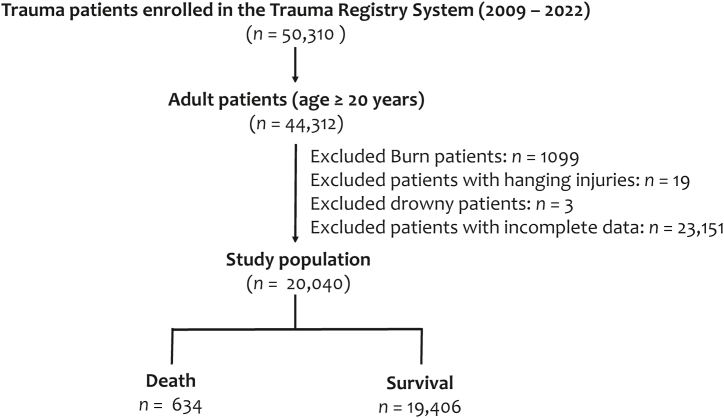
The study population enrolled from the Trauma Registry System with the patients being allocated into groups based on whether they survived or died.

The procedure was granted authorization by the Institutional Review Board (IRB) of 10.13039/100012553Chang Gung Memorial Hospital prior to the commencement of the research with the approval number 202400311B0. Specific patient consent was waived as a result of the retrospective nature of this research. As shown in Fig. 1, we enrolled 50,310 adult hospitalized patients who were equivalent to or exceeding 20 years and registered in the hospital's Trauma Registration System [21] between January 1, 2009, and December 31, 2022. These patients suffered from any type of trauma, including motor vehicle accidents (car crashes, motorcycle collisions, and pedestrian impacts), falls (from heights or on level surfaces), assaults and violent acts, sports and recreational injuries, workplace accidents, crush injuries, animal attacks or bites, and blast injuries from explosions. Those who had burned (*n* = 1099), suffered hanging injuries (*n* = 19), and drowned (*n* = 3), which mechanisms were different from the common trauma injuries, were excluded. Those patients had incomplete registered data were excluded. Finally, the study population comprised 20,040 adult patients who had sustained traumatic injuries. The medical records of patients comprise the following: demographic details (e.g., sex, age, and preexisting conditions such as hypertension [HTN], cerebrovascular accident [CVA], coronary artery disease [CAD], congestive heart failure [CHF], and diabetes mellitus [DM]), serum glucose level (mg/dL), and potassium level (mEq/L) at the time of presentation at the emergency room, the conscious level as measured by the Glasgow Coma Scale (GCS) score, the severity of the injury as assessed by the Injury Severity Score (ISS), the in-hospital mortality, and hospital stays in days.

### 2.2 Statistical analyses

The statistical software utilized for all analyses was Windows SPSS version 23.1 (IBM Inc., Chicago, IL, USA). In order to analyze categorical data, the two-sided Fisher's exact test was applied. The odds ratio (ORs) was computed incorporating a confidence interval (CI) of 95 %. The Mann-Whitney *U* test was utilized to analyze continuous data that did not follow a normal distribution. The test results were presented as medians along with interquartile ranges (IQR) spanning the first and third quartiles (Q1-Q3). Analysis of variance with Bonferroni post-hoc correction was utilized to compare continuous data to a normal distribution. The results were presented in the form of mean values and standard deviation. In order to examine the determinants of patient mortality, multivariate logistic regression was applied to univariate predictive variables. Utilizing the area under the curve (AUC) produced by the receiver operating characteristic curve (ROC), the predictive accuracy of SI for patient mortality was evaluated. Using the maximum Youden index derived from the ROC curve (sensitivity + specificity - 1) as a guide, the appropriate SI cutoff value was determined. On the basis of this ideal SI cutoff point, patients were divided into two groups, and their mortality risk was evaluated using adjusted odds ratios (AOR) with 95 % confidence intervals (CI), after controlling for significant differences in patient characteristics between the two groups. P-values with two tails, equal to 0.05, were deemed significant.

## 3 Results

### 3.1 Injury and patient characteristics between the death and survival patients

The data provided in Table 1 provide essential insight into the demographics and clinical characteristics of trauma patients, differentiating between the survivors (*n* = 19,406) and the deceased (*n* = 634). The died group had a greater percentage of men (67.0 %) compared to the survivor group (54.2 %), indicating that trauma outcomes may be more vulnerable to sex-specific factors. The mean age was notably greater in the died cohort (62.5 years) in comparison to the survivors (56.3 years), suggesting that age may be a contributing factor in trauma-related death. Furthermore, persons who did not survive had a significantly higher SI (59.7 ± 30.6 vs. 39.5 ± 17.5, p < 0.001), higher glucose level (210.7 ± 96.7 vs. 147.5 ± 61.8 mg/dL, p < 0.001), but lower potassium level (3.7 ± 0.9 vs. 3.9 ± 1.8 mEq/L, p = 0.047) compared to those who survived. In relation to pre-existing health issues, the patients who died had significantly greater rates of comorbidities such as CAD, DM, ESRD, and HTN compared to those who survived. The deceased patients had significantly lower GCS scores (median [IQR, Q1-Q3], 6 [[3], [4], [5], [6], [7], [8], [9], [10], [11], [12], [13], [14]]) but higher ISS (25 [[16], [17], [18], [19], [20], [21], [22], [23], [24], [25], [26], [27], [28], [29]]) compared to the patients who survived (GCS: 15 [15], ISS: 9 [[4], [5], [6], [7], [8], [9], [10], [11], [12], [13]], both p < 0.001).

**Table 1:** The injury and patient characteristics of patients who was deceased or survived.

Variables	Deceased *n* = 634	Survivors *n* = 19,406	OR (95 % CI)	P
Sex				<0.001
Male, *n* (%)	425(67.0)	10,519(54.2)	1.72(1.45–2.03)	
Female, *n* (%)	209(33.0)	8887(45.8)	0.58(0.49–0.69)	
Age, years	62.5 ± 18.9	56.3 ± 19.1	–	<0.001
Stress index	59.7 ± 30.6	39.5 ± 17.5	–	<0.001
Sugar (mg/dL)	210.7 ± 96.7	147.5 ± 61.8	–	<0.001
Potassium (mEq/L)	3.7 ± 0.9	3.9 ± 1.8	–	0.047
Comorbidities				
CAD, *n* (%)	69(10.9)	903(4.7)	2.50(1.93–3.24)	<0.001
CHF, *n* (%)	8(1.3)	154(0.8)	1.60(0.78–3.27)	0.195
CVA, *n* (%)	28(4.4)	892(4.6)	0.96(0.65–1.41)	0.831
DM, *n* (%)	141(22.2)	3589(18.5)	1.26(1.04–1.53)	0.017
ESRD, *n* (%)	48(7.6)	413(2.1)	3.77(2.76–5.14)	<0.001
HTN, *n* (%)	252(39.7)	6396(33.0)	1.34(1.14–1.58)	<0.001
GCS, median (IQR)	6(3–14)	15(15–15)	–	<0.001
3-8	399(62.9)	827(4.3)	38.14(32.00–45.47)	<0.001
9-12	55(8.7)	795(4.1)	2.22(1.67–2.96)	<0.001
13-15	180(28.4)	17,784(91.6)	0.04(0.03–0.04)	<0.001
ISS, median (IQR)	25(16–29)	9(4–13)	–	<0.001
1-15	90(14.2)	15,267(78.7)	0.05(0.04–0.06)	<0.001
16-24	125(19.7)	3140(16.2)	1.27(1.04–1.55)	0.018
≥25	419(66.1)	999(5.1)	35.91(30.10–42.83)	<0.001

CAD = coronary artery disease; CHF = congestive heart failure; CI = confidence interval; CVA = cerebral vascular accident; DM = diabetes mellitus; ESRD = end-stage renal disease; GCS = Glasgow Coma Scale; HTN = hypertension; IQR = interquartile range; ISS = injury severity score; OR = odds ratio.

### 3.2 Analysis of the risk factors for mortality

Table 2 presented the results of the study using both univariate and multivariate methods to determine the impact of different variables on the probability of death. Sex, age, SI, potassium and glucose levels, the preexisting CAD, DM, ESRD, or HTN, the GCS score, and the ISS were all included in this list of univariate analysis. SI (OR, 4.65; 95 % CI, 2.61–8.28; p < 0.001) was shown to be a statistically significant independent risk factor for death in multivariate logistic regression analysis. Among the SI components, glucose level (OR, 1.23; 95 % CI, 1.02–1.49; p = 0.028), but not potassium level (OR, 1.00; 95 % CI, 0.93–1.07; p = 0.976) showed statistically significant risk for death. In addition, male (OR, 1.71; 95 % CI, 1.40–2.08; p < 0.001), age (OR, 1.03; 95 % CI, 1.02–1.04; p < 0.001), the presence of CAD, DM, or ESRD, and ISS (OR, 1.16; 95 % CI, 1.15–1.17; p < 0.001) were significant independent risk factors for mortality in this study. HTN did not present as a statistically significant independent risk factor for death.

**Table 2:** The univariate and multivariate analysis of the risk factors for mortality.

	Univariate analysis			Multivariate analysis		
	OR	95 % CI	P	OR	95 % CI	P
Male	1.72	(1.45–2.03)	<0.001	1.71	(1.40–2.08)	<0.001
Age	1.02	(1.01–1.02)	<0.001	1.03	(1.02–1.04)	<0.001
Stress index	18.24	(13.94–23.87)	<0.001	4.65	(2.61–8.28)	<0.001
Sugar	2.13	(1.98–2.29)	<0.001	1.23	(1.02–1.49)	0.028
Potassium	0.69	(0.60–0.70)	<0.001	1.00	(0.93–1.07)	0.976
CAD	2.50	(1.93–3.24)	<0.001	1.51	(1.10–2.05)	0.010
DM	1.26	(1.04–1.53)	0.017	0.66	(0.51–0.85)	0.001
ESRD	3.77	(2.76–5.14)	<0.001	4.34	(3.00–6.28)	<0.001
HTN	1.34	(1.14–1.58)	<0.001	0.96	(0.77–1.18)	0.671
ISS	1.17	(1.16–1.18)	<0.001	1.16	(1.15–1.17)	<0.001

CAD = coronary artery disease; CHF = congestive heart failure; CI = confidence interval; CVA = cerebral vascular accident; DM = diabetes mellitus; ESRD = end-stage renal disease; HTN = hypertension; ISS = injury severity score; OR = odds ratio.

### 3.3 The outcomes of patients grouped based on the optimal SI cutoff value

**Fig. 2:**
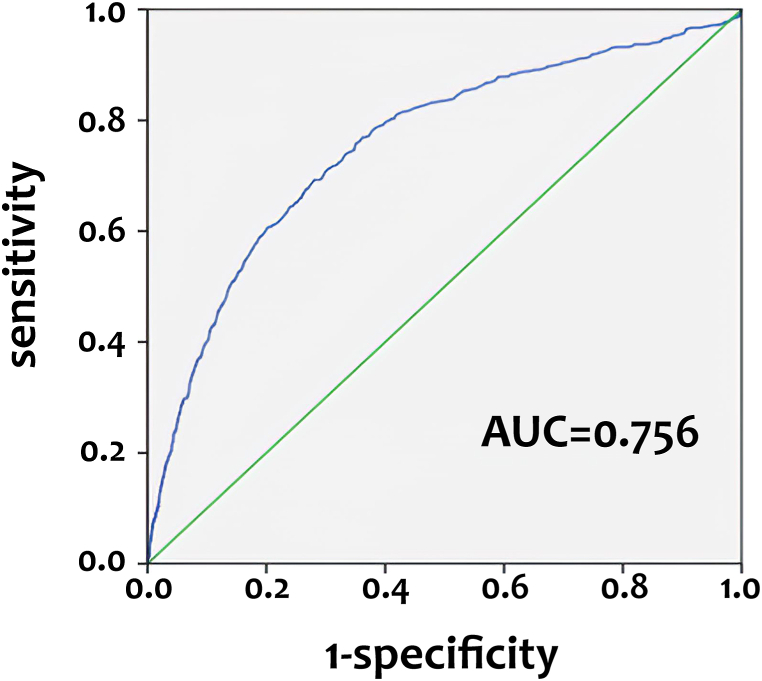
The area under the curve (AUC) and receiver operating characteristic curves (ROC) of the SI for predicting the mortality of adult trauma patients.

Based on the ROC curve analysis, a SI score of 42.7 was determined to be the ideal cutoff point, resulting in the greatest AUC value of 0.756 (Fig. 2). At this threshold, the sensitivity was 0.692 and the specificity was 0.720. Table 3 compares patients with a stress index of ≥42.7 (*n* = 5878) with those with a SI < 42.7 (*n* = 14,162) and revealed that the patients with a SI higher than or equal to 42.7 were predominantly female and significantly older than those with a SI less than 42.7. Patients with a SI of 42.7 or above had a significantly greater incidences of all examined comorbidities. Patients with a SI ≥ 42.7 had a significantly lower GCS score (13 [14,15] vs. 15 [15], p < 0.001) and a higher ISS (13 [[8], [9], [10], [11], [12], [13], [14], [15], [16], [17]] vs. 9 [[4], [5], [6], [7], [8], [9], [10]], p < 0.001) compared to those with a SI < 42.7. Patients with a SI ≥ 42.7 had a significantly longer hospital stay than those with a SI < 42.7 (12.5 vs. 8.5 days, p < 0.001). Patients with a SI ≥ 42.7 had a significantly greater death rate compared to those with a SI < 42.7 (7.5 % vs. 1.4 %, p < 0.001). After accounting for factors such as sex, age, pre-existing comorbidities, GCS, and ISS, it was shown that patients with a SI ≥ 42.7 had a significantly greater risk of death (AOR, 1.49; 95 % CI: 1.21–1.84, p < 0.001) compared to those with a SI < 42.7.

**Table 3:** The injury and patient characteristics of patients with a SI ≥ 42.7 vs. those with a SI < 42.7.

	Stress index			
Variables	≥42.7 *n* = 5878	<42.7 *n* = 14,162	OR(95 % CI)	P
Sex				<0.001
Male, *n* (%)	2971(50.5)	7973(56.3)	0.79(0.75–0.84)	
Female, *n* (%)	2907(49.5)	6189(43.7)	1.26(1.19–1.34)	
Age, years	60.8 ± 17.2	54.8 ± 19.5	–	<0.001
Comorbidities				
CAD, *n* (%)	384(6.5)	588(4.2)	1.61(1.41–1.84)	<0.001
CVA, *n* (%)	350(6.0)	570(4.0)	1.51(1.32–1.73)	<0.001
CHF, *n* (%)	60(1.0)	102(0.7)	1.42(1.03–1.96)	0.031
DM, *n* (%)	2296(39.1)	1434(10.1)	5.69(5.28–6.14)	<0.001
ESRD, *n* (%)	158(2.7)	303(2.1)	1.26(1.04–1.54)	0.018
HTN, *n* (%)	2615(44.5)	4033(28.5)	2.01(1.89–2.14)	<0.001
GCS, median (IQR)	13(14–15)	15(15–15)	–	<0.001
3-8	796(13.5)	430(3.0)	5.00(4.43–5.65)	<0.001
9-12	382(6.5)	468(3.3)	2.03(1.77–2.34)	<0.001
13-15	4700(80.0)	13,264(93.7)	0.27(0.25–0.30)	<0.001
ISS, median (IQR)	13(8–17)	9(4–10)	–	<0.001
1-15	3687(62.7)	11,670(82.4)	0.36(0.34–0.39)	<0.001
16-24	1306(22.2)	1959(13.8)	1.78(1.65–1.92)	<0.001
≥25	885(15.1)	533(3.8)	4.53(4.05–5.07)	<0.001
Hospital stays (day)	12.5 ± 13.2	8.5 ± 8.9	–	<0.001
Mortality, *n* (%)	439(7.5)	195(1.4)	5.78(4.87–6.86)	<0.001
Mortality AOR^a^	–	–	1.49(1.21–1.84)	<0.001

AOR = adjusted odds ratio; CAD = coronary artery disease; CHF = congestive heart failure; CI = confidence interval; CVA = cerebral vascular accident; DM = diabetes mellitus; ESRD = end-stage renal disease; GCS = Glasgow Coma Scale; HTN = hypertension; IQR = interquartile range; ISS = injury severity score; OR = odds ratio.

a Adjusted by sex, age, comorbidities, GCS, and ISS.

### 3.4 The outcomes of subpopulation patients grouped based on the optimal SI cutoff value

As shown in Table 4, the mortality risk, represented by adjusted odds ratios (AOR), varied across different subpopulations. Male and female patients with high SI (≥42.7) exhibited a significantly greater mortality risk (AOR 2.42, p < 0.001 and 1.24, p = 0.039, respectively) than those with low SI. Both age groups (≥65 and 20–65) demonstrated increased mortality risk for high SI patients, with AORs of 1.84 (p < 0.001) and 2.09 (p < 0.001) respectively. Patients with blunt injuries and high SI had nearly double the mortality risk (AOR 1.95, p < 0.001), while those with penetrating injuries showed no significant difference (AOR 2.96, p = 0.471). Interestingly, non-diabetic patients with high SI had a higher mortality risk (AOR 2.17, p < 0.001), whereas diabetic patients showed no significant difference in mortality risk between high and low SI groups (AOR 1.29, p = 0.231). Regarding the length of stay in the hospital, patients with high SI consistently had longer hospital stays compared to those with low SI across all subpopulations.

**Table 4:** The outcome of various subpopulation cohort of patients with a SI ≥ 42.7 vs. those with a SI < 42.7.

Subpopulation	Mortality		Hospital stay	
	AOR(95 % CI)	P	High vs. Low SI (days)	P
Male	2.42(1.87–3.14)	<0.001	13.8 ± 14.2 vs. 9.0 ± 9.6	<0.001
Female	1.24(1.04–1.81)	0.039	11.2 ± 12.0 vs. 7.8 ± 7.9	<0.001
Age ≥65	1.84(1.38–2.45)	<0.001	11.3 ± 12.0 vs. 9.0 ± 9.3	<0.001
Age 20-65	2.09(1.52–2.87)	<0.001	13.6 ± 14.1 vs. 8.2 ± 8.7	<0.001
Blunt injury	1.95(1.57–2.41)	<0.001	12.6 ± 13.2 vs. 8.6 ± 9.0	<0.001
Penetration injury	2.96(0.16–56.47)	0.471	9.4 ± 12.7 vs. 5.3 ± 5.6	<0.001
DM (+)	1.29(0.85–1.96)	0.231	11.2 ± 12.2 vs. 9.5 ± 10.0	<0.001
DM (−)	2.17(1.69–2.77)	<0.001	13.4 ± 13.8 vs. 8.3 ± 8.7	<0.001

AOR = adjusted odds ratio; CI = confidence interval; DM = diabetes mellitus; SI = stress index. The adjusted odds ratio of mortality is calculated by sex, age, comorbidities, GCS, and ISS.

## 4 Discussion

This study found that adult trauma patients with a SI ≥ 42.7 had a significantly higher death rate compared to those with a SI < 42.7. Even after accounting for the diversity in starting characteristics of the research group, the adjusted mortality rate of patients with a SI ≥ 42.7 was approximately 1.5 times greater than that of those with a SI < 42.7. The SI could be used as a prognostic indicator for trauma patients injured by all trauma causes. The optimal cutoff value of SI may be different in a distinct study patient population. There is a close correlation between an unfavorable outcome and a value of SI higher than 50 in patients with a traumatic brain injury [14,19]. There were significant correlations between the poor outcomes of the patients, with the SI set at 57.14 for those patients with aneurysmal subarachnoid hemorrhage [22] and 37.8 for those patients with aneurysmal subarachnoid hemorrhage [17].

Hyperglycemia following trauma stress is a common occurrence [[23], [24], [25], [26], [27], [28]]. Stress hormones, including epinephrine, glucocorticoid, and growth hormone and glucagon, are normally released in response to trauma-induced stress [29,30]. Brain edema, mitochondrial dysfunction, and the production of free radicals are all consequences of hyperglycemia [31] Stress hyperglycemia can be present in trauma patients with or without diabetes [32]. In severe trauma cases, particularly liver, kidney, and spleen injuries, the severity of the injury correlates with increased blood glucose levels [25,33]. Stress hyperglycemia, whether superimposed on diabetes or occurring independently, significantly raises the risk of adverse events post-surgery, including systemic infections and cardiovascular events, leading to prolonged hospital stays [33].

Hypokalemia is a common complication affecting about one-third of adult trauma patients [11]. The overactivation of the Na+/K + -ATPase pump, induced by elevated catecholamine levels, results in the translocation of potassium ions from the extracellular to intracellular compartments [34]. Factors such as congestive heart failure and head injuries are significant risk factors for hypokalemia in trauma patients [35]. Severe hypokalemia increases the likelihood of cardiac arrhythmias, particularly tachycardias. Additionally, trauma patients with hypokalemia tend to have longer hospital stays compared to those without this electrolyte imbalance. The influence of low potassium levels on the outcome in patients remains debatable [16,36,37]. It has been highlighted that moderate to severe hypokalemia is a significant predictor of mortality and adverse events in trauma patients, while mild hypokalemia does not show the same impact on mortality [11]. In this study, unlike the glucose level, the potassium level was not identified as a statistically significant risk for death.

Despite the fact that hyperglycemia and hypokalemia are patient-unfavorable factors, they are merely simple surrogate indicators of poor outcomes in clinical contexts [36]. The impact of glucose levels on the patients' outcomes was influenced by various factors, such as the existence of diabetes in the patients. For example, the outcome of patients with stress hyperglycemia is worse than that of those with diabetic hyperglycemia [23,24,27,28]. In addition, this fluctuation in serum potassium levels is part of the metabolic response to injury and reflects the body's physiological adjustments following trauma. The counter effect to the potassium during trauma is still a concern, while the potential adverse impact of potassium levels during trauma remains a matter of concern. While animal models have only shown hyperkalemia, human assays have observed both hypokalemia and hyperkalemia, along with a rebound phenomenon characterized by initial hypokalemia followed by escalating serum potassium levels [38]. Furthermore, hypokalemia after trauma injury can occur due to factors like a syndrome of inappropriate antidiuretic hormone secretion, the use of diuretic, and cerebral salt wasting [39].

This study demonstrates that the SI serves as a simple and valuable prognostic tool in the risk stratification of trauma patients from all trauma causes. It has been reported that SI is useful in the risk stratification of The SI was calculated based on two components, with the glucose level being the numerator and the potassium being the denominator, to attenuate false positive test results from one indicator [14]. The SI was considered a more accurate prognostic factor than glucose and potassium themselves [14,19]. The findings of this study showed that the SI may be used as a predictive tool for trauma patients by all trauma causes upon their initial presentation to the emergency department. In the multivariate logistic regression analysis, the SI was identified as a statistically significant independent risk factor for death, with a risk for mortality that was about 4.65 times higher. The findings aligned with previous research conducted on patients with severe traumatic brain injury, demonstrating that the SI at admission was linked to unfavorable outcomes. Specifically, there was a notable linkage between SI and higher mortality, which can assist in making informed decisions regarding surgical intervention [19].

Variations in the predictive significance of the SI were discovered across some subpopulations of trauma patients, particularly those with penetrating injuries and diabetes. The type of injury could impact the physiological response of patients who have sustained significant trauma [40,41]. Blunt injuries cause widespread trauma and stress, which has a major effect on glucose and potassium metabolism, resulting in higher SI and worse outcomes, whereas penetrating injuries are more localized, and the mortality risk is dependent on immediate and accurate injury management to reduce complications such as blood loss, pneuthorax, or hollow organ perforation-related infection. In addition, several investigations have highlighted the physiological differences between diabetes and non-diabetic patients in response to significant trauma [24,26,27,42,43]. Non-diabetic individuals with high SI had a higher mortality risk because they lack pre-existing metabolic adaptations to deal with stress-induced hyperglycemia, whereas diabetic patients may already have mechanisms in place to manage elevated glucose levels, mitigating the impact of high SI on mortality [[23], [24], [25], [26], [27], [28],^43^,^44^]. Various disparities in physiological and metabolic responses among various subpopulations may emphasize the need of taking individual patient characteristics into account when employing SI as a predictive tool in trauma management.

This study demonstrates that the SI serves as a simple and valuable prognostic tool in the risk stratification of trauma patients from all trauma causes. It has been reported that SI is useful in the risk stratification of some other illnesses, like massive and non-massive pulmonary embolism [45] or acute methylxanthine intoxication [46]. The simplicity and quick obtainability of SI make it a valuable tool for early prediction and decision-making in the management of trauma patients, offering insights into the severity of the condition and necessary interventions. However, this research has several drawbacks. Firstly, the design of the retrospective study may have generated selection bias. This bias could arise from various factors, including changes in admission criteria, specific treatment protocols and emergency interventions, or data recording practices over the study period. To address these concerns and strengthen the findings, it is crucial to conduct prospective studies in the future. Such studies would allow for standardized data collection protocols, more comprehensive patient follow-up, and the ability to control for confounding variables in real-time. Prospective designs would also enable researchers to assess the stress index's predictive value at multiple time points, providing a more dynamic understanding of its prognostic capabilities. In addition, the evaluation of results may have created a selection bias by only evaluating mortality that occurs during the hospital stay and not considering long-term mortality. This is because the trauma database used for coding does not include information on long-term mortality. Furthermore, the results seen in the study participants may have been impacted by differences in treatments, damage control effectiveness, and surgical techniques. However, it is presumed that the impacts of these treatments were uniform within the group being examined. This study utilized the blood concentrations of glucose and potassium in patients upon their presentation to the emergency department. Nevertheless, the surveillance of systemic inflammatory change brought up additional concerns, since there was a lack of understanding regarding the intricate alteration of glucose and potassium levels after trauma [16]. Moreover, comprehensive treatments including sugar control, fluid resuscitation, appropriate potassium supplementation, hypoglycemic treatment, and stress response inhibitors such as β-blockers may change the SI levels [16]. Finally, the research analysis was predominantly focused on a single urban trauma center, limiting the extent to which the findings can be generalized to other geographical locations.

## 5 Conclusions

This study underscores the significance of the stress index as a prognostic marker in trauma care. High SI values upon the arrival at emergency room are associated with increased mortality in trauma patients by all trauma causes. By integrating SI into the initial evaluation protocols, healthcare professionals may better predict patient prognosis and potentially improve overall outcomes in trauma care settings.

## Funding

This study was supported by 10.13039/100012553Chang Gung Memorial Hospital Grant No. CDRPG8M0021 & CDRPG8M0022.

## Data availability

Data available on request.

## CRediT authorship contribution statement

**Pao-Jen Kuo:** Writing – review & editing, Writing – original draft, Funding acquisition. **Ching-Ya Huang:** Writing – original draft. **Shiun-Yuan Hsu:** Formal analysis. **Ching-Hua Hsieh:** Conceptualization.

## Declaration of competing interest

The authors declare the following financial interests/personal relationships which may be considered as potential competing interests: Ching-Hua Hsieh reports a relationship with 10.13039/100012553Chang Gung Memorial Hospital Kaohsiung Branch that includes: funding grants. If there are other authors, they declare that they have no known competing financial interests or personal relationships that could have appeared to influence the work reported in this paper.
