# Supplementary material for: Evaluating the prognostic value of the stress index in trauma patients
Source: Heliyon. 2024 Aug 24;10(17):e36884. doi: 10.1016/j.heliyon.2024.e36884 (PMC11388742; doi:10.1016/j.heliyon.2024.e36884)
Supplement: Multimedia component 1 [file mmc1.docx]

Supplemental Table 1. A comparison of the patient and injury characteristics of male patients with a high and low stress index.

| Male | Stress index | |  |  |
| --- | --- | --- | --- | --- |
| Variables | ≥42.7  *n* = 2,971 | <42.7  *n* = 7,973 | OR(95%CI) | P |
| Age, years | 56.1±18.0 | 50.8±19.3 | ­－ | <0.001 |
| Comorbidities |  |  |  |  |
| CAD, *n* (%) | 199(6.7) | 327(4.1) | 1.68(1.40-2.01) | <0.001 |
| CVA, *n* (%) | 164(5.5) | 351(4.4) | 1.27(1.05-1.54) | 0.014 |
| CHF, *n* (%) | 20(0.7) | 55(0.7) | 0.98(0.58-1.63) | 0.925 |
| DM, *n* (%) | 1,001(33.7) | 681(8.5) | 5.44(4.88-6.07) | <0.001 |
| ESRD, *n* (%) | 65(2.2) | 140(1.8) | 1.25(0.93-1.69) | 0.138 |
| HTN, *n* (%) | 1,086(36.6) | 1,900(23.8) | 1.84(1.68-2.02) | <0.001 |
| GCS, median (IQR) | 15(12-15) | 15(15-15) | － | <0.001 |
| 3-8 | 538(18.1) | 329(4.1) | 5.14(4.45-5.94) | <0.001 |
| 9-12 | 220(7.4) | 297(3.7) | 2.07(1.73-2.47) | <0.001 |
| 13-15 | 2,213(74.5) | 7,347(92.1) | 0.25(0.22-0.28) | <0.001 |
| ISS, median (IQR) | 13(9-20) | 9(4-13) | － | <0.001 |
| 1-15 | 1,648(55.5) | 6,305(79.1) | 0.33(0.30-0.36) | <0.001 |
| 16-24 | 762(25.6) | 1,278(16.0) | 1.81(1.63-2.00) | <0.001 |
| ≥25 | 561(18.9) | 390(4.9) | 4.53(3.95-5.19) | <0.001 |
| Hospital stays (days) | 13.8±14.2 | 9.0±9.6 | ­－ | <0.001 |
| Mortality, *n* (%) | 295(9.9) | 130(1.6) | 6.65(5.39-8.21) | <0.001 |
| Mortality AOR* | － | － | 2.42(1.87-3.14) | <0.001 |

AOR = adjusted odds ratio; CAD = coronary artery disease; CHF = congestive heart failure; CI = confidence interval; CVA = cerebral vascular accident; DM = diabetes mellitus; ESRD = end-stage renal disease; GCS = Glasgow Coma Scale; HTN = hypertension; IQR = interquartile range; ISS = injury severity score; OR= odds ratio. * adjusted by age, comorbidities, GCS, and ISS

Supplemental Table 2. A comparison of the patient and injury characteristics of female patients with a high and low stress index.

| Female | Stress index | |  |  |
| --- | --- | --- | --- | --- |
| Variables | ≥42.7  *n* = 2,907 | <42.7  *n* = 6,189 | OR(95%CI) | P |
| Age, years | 65.6±15.0 | 59.8±18.6 | ­－ | <0.001 |
| Comorbidities |  |  |  |  |
| CAD, *n* (%) | 185(6.4) | 261(4.2) | 1.54(1.27-1.87) | <0.001 |
| CVA, *n* (%) | 186(6.4) | 219(3.5) | 1.86(1.53-2.28) | <0.001 |
| CHF, *n* (%) | 40(1.4) | 47(0.8) | 1.82(1.19-2.79) | 0.005 |
| DM, *n* (%) | 1,295(44.5) | 753(12.2) | 5.80(5.22-6.45) | <0.001 |
| ESRD, *n* (%) | 93(3.2) | 163(2.6) | 1.22(0.94-1.58) | 0.128 |
| HTN, *n* (%) | 1,529(52.6) | 2,133(34.5) | 2.11(1.93-2.31) | <0.001 |
| GCS, median (IQR) | 15(15-15) | 15(15-15) | － | <0.001 |
| 3-8 | 258(8.9) | 101(1.6) | 5.87(4.64-7.42) | <0.001 |
| 9-12 | 162(5.6) | 171(2.8) | 2.08(1.67-2.59) | <0.001 |
| 13-15 | 2,487(85.6) | 5,917(95.6) | 0.27(0.23-0.32) | <0.001 |
| ISS, median (IQR) | 9(5-16) | 9(4-9) | － | <0.001 |
| 1-15 | 2,039(70.1) | 5,365(86.7) | 0.36(0.32-0.40) | <0.001 |
| 16-24 | 544(18.7) | 681(11.0) | 1.86(1.65-2.11) | <0.001 |
| ≥25 | 324(11.1) | 143(2.3) | 5.30(4.33-6.49) | <0.001 |
| Hospital stays (days) | 11.2±12.0 | 7.8±7.9 | ­－ | <0.001 |
| Mortality, *n* (%) | 144(5.0) | 65(1.1) | 4.91(3.65-6.60) | <0.001 |
| Mortality AOR* | － | － | 1.24(1.04-1.81) | 0.039 |

AOR = adjusted odds ratio; CAD = coronary artery disease; CHF = congestive heart failure; CI = confidence interval; CVA = cerebral vascular accident; DM = diabetes mellitus; ESRD = end-stage renal disease; GCS = Glasgow Coma Scale; HTN = hypertension; IQR = interquartile range; ISS = injury severity score; OR= odds ratio. * adjusted by age, comorbidities, GCS, and ISS

Supplemental Table 3. A comparison of the patient and injury characteristics of elderly patients with a high and low stress index.

| Age≥65 | Stress index | |  |  |
| --- | --- | --- | --- | --- |
| Variables | ≥42.7  *n* = 2,721 | <42.7  *n* = 4,838 | OR(95%CI) | P |
| Sex |  |  |  | <0.001 |
| Male, *n* (%) | 1,043(38.3) | 2,120(43.8) | 0.80(0.72-0.88) |  |
| Female, *n* (%) | 1,678(61.7) | 2,718(56.2) | 1.26(1.14-1.38) |  |
| Comorbidities |  |  |  |  |
| CAD, *n* (%) | 301(11.1) | 464(9.6) | 1.17(1.01-1.37) | 0.042 |
| CVA, *n* (%) | 273(10.0) | 457(9.4) | 1.07(0.91-1.25) | 0.407 |
| CHF, *n* (%) | 44(1.6) | 74(1.5) | 1.06(0.73-1.54) | 0.768 |
| DM, *n* (%) | 1,419(52.1) | 960(19.8) | 4.40(3.97-4.88) | <0.001 |
| ESRD, *n* (%) | 94(3.5) | 194(4.0) | 0.86(0.67-1.10) | 0.226 |
| HTN, *n* (%) | 1,749(64.3) | 2,709(56.0) | 1.41(1.28-1.56) | <0.001 |
| GCS, median (IQR) | 15(15-15) | 15(15-15) | － | <0.001 |
| 3-8 | 271(10.0) | 122(2.5) | 4.28(3.43-5.32) | <0.001 |
| 9-12 | 151(5.5) | 186(3.8) | 1.47(1.18-1.83) | 0.001 |
| 13-15 | 2,299(84.5) | 4,530(93.6) | 0.37(0.32-0.43) | <0.001 |
| ISS, median (IQR) | 9(9-16) | 9(5-13) | － | <0.001 |
| 1-15 | 1,804(66.3) | 3,781(78.2) | 0.55(0.50-0.61) | <0.001 |
| 16-24 | 586(21.5) | 848(17.5) | 1.29(1.15-1.45) | <0.001 |
| ≥25 | 331(12.2) | 209(4.3) | 3.07(2.56-3.67) | <0.001 |
| Hospital stays (days) | 11.3±12.0 | 9.0±9.3 | － | <0.001 |
| Mortality, *n* (%) | 220(8.1) | 113(2.3) | 3.68(2.92-4.64) | <0.001 |
| Mortality AOR* | － | － | 1.84(1.38-2.45) | <0.001 |

AOR = adjusted odds ratio; CAD = coronary artery disease; CHF = congestive heart failure; CI = confidence interval; CVA = cerebral vascular accident; DM = diabetes mellitus; ESRD = end-stage renal disease; GCS = Glasgow Coma Scale; HTN = hypertension; IQR = interquartile range; ISS = injury severity score; OR= odds ratio. * adjusted by sex, comorbidities, GCS, and ISS

Supplemental Table 4. A comparison of the patient and injury characteristics of adult patients aged of 20-64 years with a high and low stress index.

| Age 20-64 | Stress index | |  |  |
| --- | --- | --- | --- | --- |
| Variables | ≥42.7  *n* = 3,157 | <42.7  *n* = 9,324 | OR(95%CI) | P |
| Sex |  |  |  | 0.088 |
| Male, *n* (%) | 1,928(61.1) | 5,853(62.8) | 0.93(0.86-1.01) |  |
| Female, *n* (%) | 1,229(38.9) | 3,471(37.2) | 1.08(0.99-1.17) |  |
| Comorbidities |  |  |  |  |
| CAD, *n* (%) | 83(2.6) | 124(1.3) | 2.00(1.51-2.65) | <0.001 |
| CVA, *n* (%) | 77(2.4) | 113(1.2) | 2.04(1.52-2.73) | <0.001 |
| CHF, *n* (%) | 16(0.5) | 28(0.3) | 1.69(0.91-3.13) | 0.091 |
| DM, *n* (%) | 877(27.8) | 474(5.1) | 7.18(6.36-8.10) | <0.001 |
| ESRD, *n* (%) | 64(2.0) | 109(1.2) | 1.75(1.28-2.39) | <0.001 |
| HTN, *n* (%) | 866(27.4) | 1,324(14.2) | 2.28(2.07-2.52) | <0.001 |
| GCS, median (IQR) | 15(13-15) | 15(15-15) | － | <0.001 |
| 3-8 | 525(16.6) | 308(3.3) | 5.84(5.04-6.77) | <0.001 |
| 9-12 | 231(7.3) | 282(3.0) | 2.53(2.12-3.03) | <0.001 |
| 13-15 | 2,401(76.1) | 8,734(93.7) | 0.22(0.19-0.24) | <0.001 |
| ISS, median (IQR) | 10(5-20) | 4(4-9) | － | <0.001 |
| 1-15 | 1,883(59.6) | 7,889(84.6) | 0.27(0.25-0.29) | <0.001 |
| 16-24 | 720(22.8) | 1,111(11.9) | 2.18(1.97-2.42) | <0.001 |
| ≥25 | 554(17.5) | 324(3.5) | 5.91(5.12-6.83) | <0.001 |
| Hospital stays (days) | 13.6±14.1 | 8.2±8.7 | － | <0.001 |
| Mortality, *n* (%) | 219(6.9) | 82(0.9) | 8.40(6.50-10.87) | <0.001 |
| Mortality AOR* | － | － | 2.09(1.52-2.87) | <0.001 |

AOR = adjusted odds ratio; CAD = coronary artery disease; CHF = congestive heart failure; CI = confidence interval; CVA = cerebral vascular accident; DM = diabetes mellitus; ESRD = end-stage renal disease; GCS = Glasgow Coma Scale; HTN = hypertension; IQR = interquartile range; ISS = injury severity score; OR= odds ratio. * adjusted by sex, comorbidities, GCS, and ISS

Supplemental Table 5. A comparison of the patient and injury characteristics of blunt injured patients with a high and low stress index.

| Blunt | Stress index | |  |  |
| --- | --- | --- | --- | --- |
| Variables | ≥42.7  *n* = 5,761 | <42.7  *n* = 13,580 | OR(95%CI) | P |
| Sex |  |  |  | <0.001 |
| Male, *n* (%) | 2,881(50.0) | 7,525(55.4) | 0.81(0.76-0.86) |  |
| Female, *n* (%) | 2,880(50.0) | 6,055(44.6) | 1.24(1.17-1.32) |  |
| Age, years | 61.0±17.2 | 55.1±19.6 | ­－ | <0.001 |
| Comorbidities |  |  |  |  |
| CAD, *n* (%) | 381(6.6) | 580(4.3) | 1.59(1.39-1.81) | <0.001 |
| CVA, *n* (%) | 349(6.1) | 566(4.2) | 1.48(1.29-1.70) | <0.001 |
| CHF, *n* (%) | 59(1.0) | 101(0.7) | 1.38(1.00-1.91) | 0.049 |
| DM, *n* (%) | 2,269(39.4) | 1,409(10.4) | 5.61(5.20-6.06) | <0.001 |
| ESRD, *n* (%) | 156(2.7) | 300(2.2) | 1.23(1.01-1.50) | 0.037 |
| HTN, *n* (%) | 2,578(44.7) | 3,964(29.2) | 1.97(1.84-2.09) | <0.001 |
| GCS, median (IQR) | 15(14-15) | 15(15-15) | － | <0.001 |
| 3-8 | 784(13.6) | 428(3.2) | 4.84(4.28-5.47) | <0.001 |
| 9-12 | 376(6.5) | 465(3.4) | 1.97(1.71-2.26) | <0.001 |
| 13-15 | 4,601(79.9) | 12,687(93.4) | 0.28(0.25-0.31) | <0.001 |
| ISS, median (IQR) | 9(9-17) | 9(4-10) | － | <0.001 |
| 1-15 | 3,582(62.2) | 11,094 | 0.37(0.34-0.40) | <0.001 |
| 16-24 | 1,301(22.6) | 1,953(14.4) | 1.74(1.61-1.88) | <0.001 |
| ≥25 | 878(15.2) | 533(3.9) | 4.40(3.93-4.93) | <0.001 |
| Hospital stays (days) | 12.6±13.2 | 8.6±9.0 | － | <0.001 |
| Mortality, *n* (%) | 432(7.5) | 195(1.4) | 5.56(4.69-6.61) | <0.001 |
| Mortality AOR* | － | － | 1.95(1.57-2.41) | <0.001 |

AOR = adjusted odds ratio; CAD = coronary artery disease; CHF = congestive heart failure; CI = confidence interval; CVA = cerebral vascular accident; DM = diabetes mellitus; ESRD = end-stage renal disease; GCS = Glasgow Coma Scale; HTN = hypertension; IQR = interquartile range; ISS = injury severity score; OR= odds ratio. * adjusted by sex, age, comorbidities, GCS, and ISS

Supplemental Table 6. A comparison of the patient and injury characteristics of penetration-injuried patients with a high and low stress index.

| Penetrating | Stress index | |  |  |
| --- | --- | --- | --- | --- |
| Variables | ≥42.7  *n* = 111 | <42.7  *n* = 588 | OR(95%CI) | P |
| Sex |  |  |  | 0.889 |
| Male, *n* (%) | 86(77.5) | 452(76.9) | 1.04(0.64-1.68) |  |
| Female, *n* (%) | 25(22.5) | 136(23.1) | 0.97(0.60-1.57) |  |
| Age, years | 50.7±14.7 | 45.6±15.8 | ­－ | 0.002 |
| Comorbidities |  |  |  |  |
| CAD, *n* (%) | 3(2.7) | 8(1.4) | 2.01(0.53-7.71) | 0.297 |
| CVA, *n* (%) | 0(0.0) | 5(0.9) | － | － |
| CHF, *n* (%) | 1(0.9) | 1(0.2) | 5.34(0.33-85.96) | 0.186 |
| DM, *n* (%) | 27(24.3) | 25(4.3) | 7.24(4.01-13.06) | <0.001 |
| ESRD, *n* (%) | 2(1.8) | 3(0.5) | 3.58(0.59-21.66) | 0.139 |
| HTN, *n* (%) | 34(30.6) | 72(12.2) | 3.17(1.97-5.08) | <0.001 |
| GCS, median (IQR) | 15(15-15) | 15(15-15) | － | <0.001 |
| 3-8 | 10(9.0) | 4(0.7) | 14.46(4.45-46.98) | <0.001 |
| 9-12 | 5(4.5) | 4(0.7) | 6.89(1.82-26.06) | 0.001 |
| 13-15 | 96(86.5) | 580(98.6) | 0.09(0.04-0.21) | <0.001 |
| ISS, median (IQR) | 4(4-5) | 4(1-4) | － | <0.001 |
| 1-15 | 101(91.0) | 580(89.6) | 0.14(0.05-0.36) | <0.001 |
| 16-24 | 4(3.6) | 7(1.2) | 3.10(0.89-10.78) | 0.061 |
| ≥25 | 6(5.4) | 1(0.2) | 33.54(4.00-281.46) | <0.001 |
| Hospital stays (days) | 9.4±12.7 | 5.3±5.6 | － | <0.001 |
| Mortality, *n* (%) | 6(5.4) | 1(0.2) | 33.54(4.00-281.45) | <0.001 |
| Mortality AOR* | － | － | 2.96(0.16-56.47) | 0.471 |

AOR = adjusted odds ratio; CAD = coronary artery disease; CHF = congestive heart failure; CI = confidence interval; CVA = cerebral vascular accident; DM = diabetes mellitus; ESRD = end-stage renal disease; GCS = Glasgow Coma Scale; HTN = hypertension; IQR = interquartile range; ISS = injury severity score; OR= odds ratio. * adjusted by sex, age, comorbidities, GCS, and ISS

Supplemental Table 7. A comparison of the patient and injury characteristics of diabetic patients with a high and low stress index.

| DM(+) | Stress index | |  |  |
| --- | --- | --- | --- | --- |
| Variables | ≥42.7  *n* = 2,296 | <42.7  *n* = 1,434 | OR(95%CI) | P |
| Sex |  |  |  | 0.020 |
| Male, *n* (%) | 1,001(43.6) | 681(47.5) | 0.86(0.75-0.98) |  |
| Female, *n* (%) | 1,295(56.4) | 753(52.5) | 1.17(1.03-1.34) |  |
| Age, years | 67.4±12.2 | 69.3±11.9 | ­－ | <0.001 |
| Comorbidities |  |  |  |  |
| CAD, *n* (%) | 242(10.5) | 175(12.2) | 0.85(0.69-1.04) | 0.117 |
| CVA, *n* (%) | 223(9.7) | 164(11.4) | 0.83(0.67-1.03) | 0.093 |
| CHF, *n* (%) | 45(2.0) | 26(1.8) | 1.08(0.67-1.76) | 0.750 |
| ESRD, *n* (%) | 123(5.4) | 103(7.2) | 0.73(0.56-0.96) | 0.023 |
| HTN, *n* (%) | 1,548(67.4) | 1,042(72.7) | 0.78(0.67-0.90) | 0.001 |
| GCS, median (IQR) | 15(15-15) | 15(15-15) | － | <0.001 |
| 3-8 | 132(5.7) | 33(2.3) | 2.59(1.76-3.82) | <0.001 |
| 9-12 | 103(4.5) | 60(4.2) | 1.08(0.78-1.49) | 0.661 |
| 13-15 | 2,061(89.8) | 1,341(93.5) | 0.61(0.47-0.78) | <0.001 |
| ISS, median (IQR) | 9(5-16) | 9(4-13) | － | <0.001 |
| 1-15 | 1,677(73.0) | 1,116(77.8) | 0.77(0.66-0.90) | 0.001 |
| 16-24 | 439(19.1) | 262(18.3) | 1.06(0.89-1.25) | 0.518 |
| ≥25 | 180(7.8) | 56(3.9) | 2.09(1.54-2.85) | <0.001 |
| Hospital stays (days) | 11.2±12.2 | 9.5±10.0 | － | <0.001 |
| Mortality, *n* (%) | 101(4.4) | 40(2.8) | 1.60(1.11-2.33) | 0.012 |
| Mortality AOR* | － | － | 1.29(0.85-1.96) | 0.231 |

AOR = adjusted odds ratio; CAD = coronary artery disease; CHF = congestive heart failure; CI = confidence interval; CVA = cerebral vascular accident; DM = diabetes mellitus; ESRD = end-stage renal disease; GCS = Glasgow Coma Scale; HTN = hypertension; IQR = interquartile range; ISS = injury severity score; OR= odds ratio. * adjusted by sex, age, comorbidities, GCS, and ISS

Supplemental Table 8. A comparison of the patient and injury characteristics of non-diabetic patients with a high and low stress index.

| DM(-) | Stress index | |  |  |
| --- | --- | --- | --- | --- |
| Variables | ≥42.7  *n* = 3,582 | <42.7  *n* = 12,728 | OR(95%CI) | P |
| Sex |  |  |  | 0.014 |
| Male, *n* (%) | 1,970(55.0) | 7,292(57.3) | 0.91(0.85-0.98) |  |
| Female, *n* (%) | 1,612(45.0) | 5,436(42.7) | 1.10(1.02-1.18) |  |
| Age, years | 56.6±18.6 | 53.1±19.5 | ­－ | <0.001 |
| Comorbidities |  |  |  |  |
| CAD, *n* (%) | 142(4.0) | 413(3.2) | 1.23(1.01-1.50) | 0.036 |
| CVA, *n* (%) | 127(3.5) | 406(3.2) | 1.12(0.91-1.37) | 0.290 |
| CHF, *n* (%) | 15(0.4) | 76(0.6) | 0.70(0.40-1.22) | 0.206 |
| ESRD, *n* (%) | 35(1.0) | 200(1.6) | 0.62(0.43-0.89) | 0.008 |
| HTN, *n* (%) | 1,067(29.8) | 2,991(23.5) | 1.38(1.27-1.50) | <0.001 |
| GCS, median (IQR) | 15(12-15) | 15(15-15) | － | <0.001 |
| 3-8 | 664(18.5) | 397(3.1) | 7.07(6.20-8.06) | <0.001 |
| 9-12 | 279(7.8) | 408(3.2) | 2.55(2.18-2.98) | <0.001 |
| 13-15 | 2,639(73.7) | 11,923(93.7) | 0.19(0.17-0.21) | <0.001 |
| ISS, median (IQR) | 13(9-20) | 8(4-9) | － | <0.001 |
| 1-15 | 2010(56.1) | 10554(82.9) | 0.26(0.24-0.29) | <0.001 |
| 16-24 | 867(24.2) | 1697(13.3) | 2.08(1.89-2.28) | <0.001 |
| ≥25 | 705(19.7) | 477(3.7) | 6.29(5.57-7.12) | <0.001 |
| Hospital stays (day) | 13.4±13.8 | 8.3±8.7 | － | <0.001 |
| Mortality, *n* (%) | 338(9.4) | 155(1.2) | 8.45(6.96-10.26) | <0.001 |
| Mortality AOR* | － | － | 2.17(1.69-2.77) | <0.001 |

AOR = adjusted odds ratio; CAD = coronary artery disease; CHF = congestive heart failure; CI = confidence interval; CVA = cerebral vascular accident; DM = diabetes mellitus; ESRD = end-stage renal disease; GCS = Glasgow Coma Scale; HTN = hypertension; IQR = interquartile range; ISS = injury severity score; OR= odds ratio. * adjusted by sex, age, comorbidities, GCS, and ISS
